# Exploring Behavior of People with Suicidal Ideation in a Chinese Online Suicidal Community

**DOI:** 10.3390/ijerph16010054

**Published:** 2018-12-26

**Authors:** Zheng Wang, Guang Yu, Xianyun Tian

**Affiliations:** School of Management, Harbin Institute of Technology, Harbin 150001, China; wzxdmail@gmail.com (Z.W.); uncertainorcertain@gmail.com (X.T.)

**Keywords:** social media, suicide, Weibo, people with suicidal ideation, suicidal community

## Abstract

People with suicidal ideation (PSI) are increasingly using social media to express suicidal feelings. Researchers have found that their internet-based communication may lead to the spread of suicidal ideation, which presents a set of challenges for suicide prevention. To develop effective prevention and intervention strategies that can be efficiently applied in online communities, we need to understand the behavior of PSI in internet-based communities. However, to date there have been no studies that specifically focus on the behavior of PSI in Chinese online communities. A total of 4489 postings in which users explicitly expressed their suicidal ideation were labeled from 560,000 postings in an internet-based suicidal community on Weibo (one of the biggest social media platforms in China) to explore their behavior. The results reveal that PSI are significantly more active than other users in the community. With the use of social network analysis, we also found that the more frequently users communicate with PSI, the more likely that users would become suicidal. In addition, Chinese women may be more likely to be at risk of suicide than men in the community. This study enriches our knowledge of PSI’s behavior in online communities, which may contribute to detecting and assisting PSI on social media.

## 1. Introduction

Sina Weibo (Weibo), Chinese version of Twitter, is one of the most popular social media platforms among the Chinese population [1]. Like Twitter, Weibo allows its users to post updates and comment on updates which were posted by other users. Moreover, many active users may comment on mental health-related updates [2,3], and some of them may be troubled by self-reported health problems, such as depression and suicide [4,5].

Suicide is a severe public health problem around the world and is particularly serious in China, which accounts for more than 30% of the world’s suicides [6,7]. It has been reported that mental disorder was a less important risk factor for suicide in China [8]. Other factors, such as acute stressors and impulsivity, might play a more important role in Chinese suicides (Chinese women are more likely to commit suicide [9]) than in Western suicides [8,10,11]. As impulsive suicide attempts are hard to predict, it is a challenge to intervene in a timely manner [12,13,14]. Fortunately, the emergence of social media (including Facebook and Twitter) provides a new pathway for helping people with suicidal ideation (PSI) [15,16]. Researchers have demonstrated that suicidal ideation can be disclosed by suicide-related words and phrases on social media [17]. For example, a 21-year-old Sweden man died by hanging hours after posting some suicide-related words on an internet forum in 2010, and there are more than 3000 comments under this suicidal posting [18]. With the use of suicide-related words, PSI can be detected on social media effectively and their suicide attempts can be prevented [19,20,21].

It is worth noting that PSI can also use suicide-related words to search for users who are similar to themselves on social media [22,23,24,25]. In this way, PSI get acquainted with each other, and then cluster together in so-called “suicidal communities” (where the people participating are primarily PSI) to share their suicidal feelings, ideation or plans anonymously [26,27,28]. That is, the existence of suicidal communities may facilitate communication among PSI. Although their internet-based communication in the community is reported as a way to reduce the pressure to take suicidal action [29], their communication may lead to the spread of suicide methods, ideation and deaths [30,31], which present a set of challenges for suicide prevention [32]. Koburger [33] analyzed a significant increase of railway suicides after a report of a railway suicide, and concluded that the spread of suicide methods may trigger copycat suicides. Joiner [32] explored the communication of people who are vulnerable to suicide in a suicidal community, and found that all members of the community may be at increased risk for suicidality due to the spread of suicidal ideation. By studying the formation and expansion of the suicidal community, Johansson et al. [28] were able to demonstrate that PSI in the community might super-infect one another (e.g., some PSI are highly motivated to find partners to commit suicide [23]), and then more vulnerable users will be attracted to join in from the community. In other words, if effective measures are not taken to prevent suicides, the community may become a hotbed of suicide.

In China, social media platforms are increasingly common platforms for people to broadcast suicidal ideation [34], and online suicidal communities do exist in China (as far as we know, the size of online suicidal communities vary greatly, and there is only one community with more than 100,000 participants) [3,35]. As PSI are very likely to tell others about their suicidal thoughts on Weibo, researchers are trying to detect and help PSI according to their postings on Weibo [36]. For example, by studying the postings PSI published on Weibo, Wang et al. [37] were able to conclude that PSI are more active at night, and more women than men report their suicidal ideation. Some researchers explored the linguistic features of PSI on Weibo, and found that PSI may use less verbs, and post more words in their updates [38]. However, these studies have mainly focused on exploring their behavior at an individual level [39,40], and to date there have been no studies that specifically focus on the behavior of PSI in online suicidal communities. That is, we have limited knowledge of the behavior of Chinese PSI in online communities.

This study addresses this knowledge gap by identifying and describing PSI in a suicidal community [35], so that better detection and intervention measures can be adopted to assist them. Specifically, the study classifies the themes of suicidal comments published by PSI, examines whether and how suicidal ideation spread in the community, identifies the characteristics of PSI, and explores the active patterns of individuals who may be at risk of committing suicide.

## 2. Materials and Methods

The data used in this study are public. To protect the privacy of the individuals, we removed the identity information of users.

### 2.1. Data Collection

The suicidal community we studied exists in the comments of a Chinese college student’s last posting before the student committed suicide. This student, posted on Weibo with the username “Zoufan”, committed suicide on March 17, 2012, just after leaving a farewell posting that auto-posted online hours after her death [3,35]. For the past six years, there have been over 560,000 comments under the farewell posting and the number of comments is still continuing to grow.

**Figure 1:**
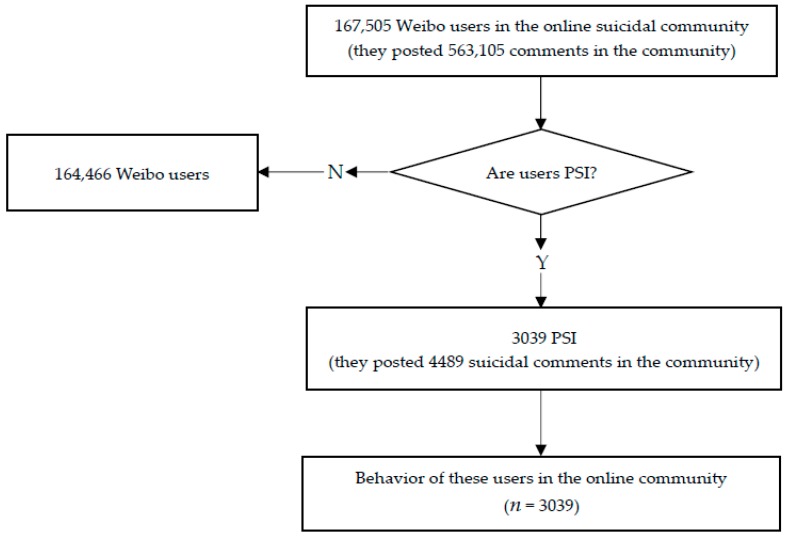
Flow chart of the study.

From January 17 to February 18, 2017, all the comments of Zoufan’s last posting, approximately 560 thousand comments by 160 thousand users, were crawled from Weibo with the use of a spider programmed by the research team. Along with these comments, demographic characteristics of these users were also obtained. As these data are still changing, we updated the demographic characteristics of these 160 thousand users from September 29 to October 8, 2018. The flow chart of the data processing is presented in Figure 1.

### 2.2. Coding of Suicidal Comments

Considering the fact that suicidality was self-reported rather than observed by others in most clinical trials [41,42], human coding is used to determine whether the user has suicidal ideation (the definition of suicidal ideation refers to thoughts of engaging in behavior intended to end one’s life [43]) in his/her suicide-related comments [44,45].

Comments in which users explicitly expressed their suicidal ideation were identified as suicidal comments (e.g., kill myself; end my life), and other comments, including those in which users did not explicitly express their suicidal ideation, were discarded (e.g., I don’t want to live, but don’t want to die either). To accurately identify suicidal comments, human coding (which was employed by three mental health researchers (the authors) who specialized in suicide prevention and possessed training in the detection of suicide risk) was used to determine whether comments are suicidal ones. A three step coding process was employed as follows: The first step was to ask two of the researchers to detect suicidal comments individually and code according to a classification system reiterated by the research team; the second step required them to reach an agreement on differences by consensus; and in the final step the third researcher was asked to code a sample of 2000 items (randomly selected from the dataset) in order to calculate the inter-rater reliability (kappa > 0.8 indicates a substantial agreement [46,47,48]). To avoid annotation fatigue, our researchers judged 1000 comments per hour. Then, 4489 comments were identified as suicidal ones and these comments belonged to 3039 Weibo users. The inter-rater reliability was good at kappa 0.84.

### 2.3. Coding of Themes

Themes were defined as topics that occur repeatedly [49]. To learn the themes of PSI’s suicidal comments, we scanned all these comments to determine the most popular themes. These comments were grouped into following themes: (1) expression of suicidal ideation, (2) inquiry of suicide methods, (3) expression of thanatophobia, (4) description of suicide attempts, and (5) seeking suicide partners.

The coding process was similar to the process mentioned above, after which the inter-coder reliability for each theme was calculated and tabulated as shown in Table 1. According to the magnitude guidelines proposed by Landis and Koch [46], all the kappa values in the study were acceptable.

### 2.4. Interactional Patterns

To explore the interactional patterns of PSI in the community, social network analysis is introduced to describe the communication with PSI participation.

**Figure 2:**
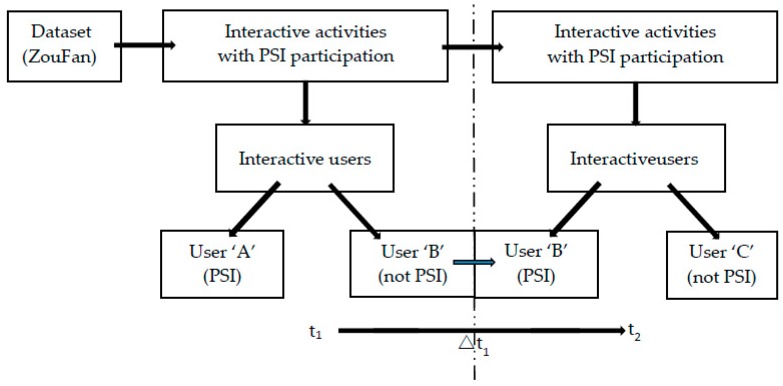
Model depicting the spread of suicidal ideation in the community.

As shown in Figure 2, if there is an association between suicidal ideation of user ‘B’ and his/her communication with PSI (e.g., user ‘A’), we consider that both user ‘A’ and user ‘B’ contribute to the spread of suicidal ideation in the community.

### 2.5. Data Analysis

Network analysis was used to depict interactional patterns of PSI in the community, and Gephi (version 0.91) [50] was used for constructing and visualizing the communication network. The difference in the number of followers, following, and postings was measured by Cohen’s d [51], Wilcoxon Rank Sum, and Signed Rank Tests [52]. The diurnal pattern of the postings was presented by ggplot2 (version 2.0.0) [53]. All the analyses were operated by Python (version 3.5) [54].

## 3. Results

### 3.1. Themes

A total of 4489 suicidal comments (posted by 3039 PSI) were labeled from 560,000 comments. Themes identified in these comments are presented in Table 2.

The majority of these suicidal comments simply expressed the suicidal ideation (73.5%, 3300/4489). The second most common theme (11.4%, 513/4489) was expression of thanatophobia. The other three themes were much less frequent. Seeking suicide partners (6.8%, 304/4489) was also discussed. Only 5.9% of the comments (263/4489) mentioned inquiry of suicide methods. The rest of the comments were descriptions of previous suicide attempts (2.6%, 117/4489). These theme statistics reveals that some PSI were highly motivated to find methods by which and partners with whom to commit suicide, although most of them only expressed suicidal ideation in their comments.

As presented in Table 2, there were 8 comments simultaneously recorded in the theme ‘Expression of suicidal ideation’ and the theme ‘Description of previous suicide attempts’, in which these PSI not only described their previous suicide attempts, but also desired to attempt to commit suicide again. This phenomenon suggests that suicide attempters may still be troubled by suicidal ideation and need to be assisted in a timely and effective manner.

### 3.2. Interactional Patterns of PSI

With the use of social network analysis, we found that there was a positive correlation between the times users communicated with PSI and the probability that users were in the same situation (the correlation coefficient is 0.98 [54,55]). That is, communicating with PSI, to some extent, may lead to the spread of suicidal ideation [56]. Then, a total of 204 PSI who were involved in the spread of suicidal ideation were detected from the 3039 PSI identified above. To examine whether there were differences in expressing suicidal ideation between PSI who were involved in the spread of suicidal ideation, and other PSI in the community, we examined the suicidal themes mentioned by the two groups. The results are shown in Table 3.

This result indicates that there was a statistically significant difference (*χ*^2^ = 77.9, *p* < 0.001) between the two groups in the theme of seeking suicide partners. The percentage of this theme mentioned by PSI involved in spreading suicidal ideation was 16.2%, while among the other PSI only 5.6% mentioned this theme.

**Figure 3:**
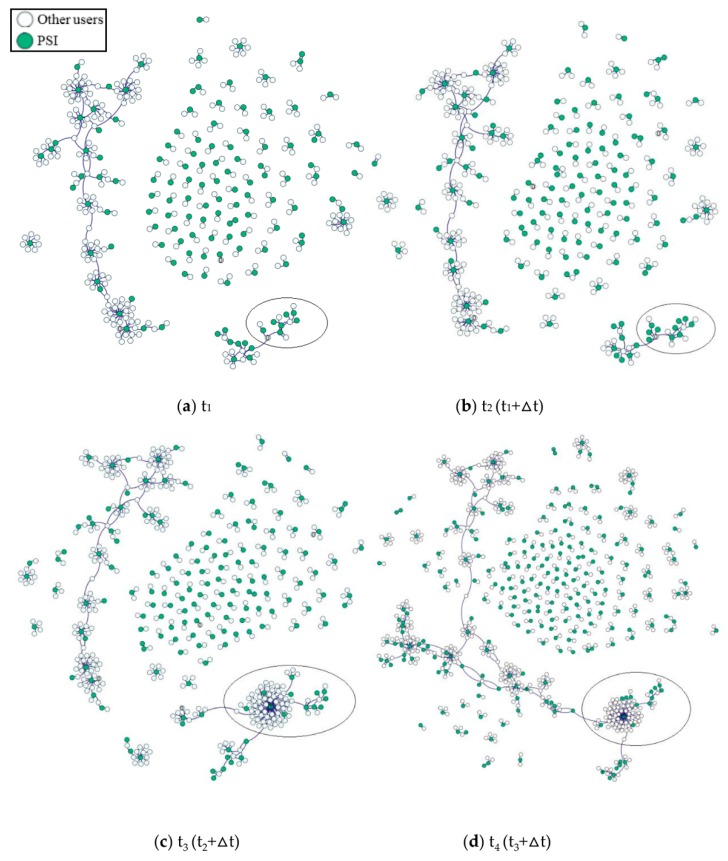
Interactional patterns of PSI. (**a**) Communication at the time t_1_; (**b**) Communication at the time t_2_; (**c**) Communication at the time t_3_; (**d**) Communication at the time t_4_. The hollow nodes in the fig represent the users who are not PSI, and the solid nodes represent PSI. The edges connecting two nodes indicate that the frequency of their communication is greater than 5 times. PSI, people with suicidal ideation.

To graphically describe the interactional patterns of PSI, the communication patterns of different moments are illustrated in Figure 3. To clearly illustrate the spread of suicidal ideation, only those users who have contacted each other more than 5 times are shown in the graph. It can be observed from Figure 3 that a user who did not have the idea of committing suicide before the initial state (labeled as “0” in the ellipse box of Figure 3a,b), expressed suicidal ideation at the following state (labeled as ‘1’ in the ellipse box of Figure 3c,d) after communicating with PSI. This result suggests that there may be suicide contagion along with the spread of suicidal ideation. In addition, the formation and expansion of a sub-community centered on this user (Figure 3c,d), indicates that suicide may be transmitted within a specific community and lead to a clustering phenomenon.

### 3.3. Demographics Characteristics of PSI

There was a total 3039 PSI detected from the users in the community (*n* = 167,505). Demographic information of the groups is presented in Table 4. As shown in the table, 75% of the PSI were female, which was significantly higher (*p* < 0.001) compared with the other users in the community (65%).

The PSI group had significantly fewer followers (*p* < 0.001), following (*p* < 0.001) and postings (*p* < 0.001) compared to the control group which are presented in Table 4. This suggests that PSI may be relatively less active on Weibo.

### 3.4. Active Patterns of PSI

To investigate the active patterns of PSI in the community, the number of users and active users who posted in the community on 3 or more days a week were counted. Results show that the number of PSI and active PSI (Table 5) in the community have increased year by year, indicating the high influence of the suicidal community and providing evidence for the agglomeration of PSI. By comparing the proportion of active users, the proportion of active PSI was significantly greater. This difference suggests that PSI may be more active in the community.

**Figure 4:**
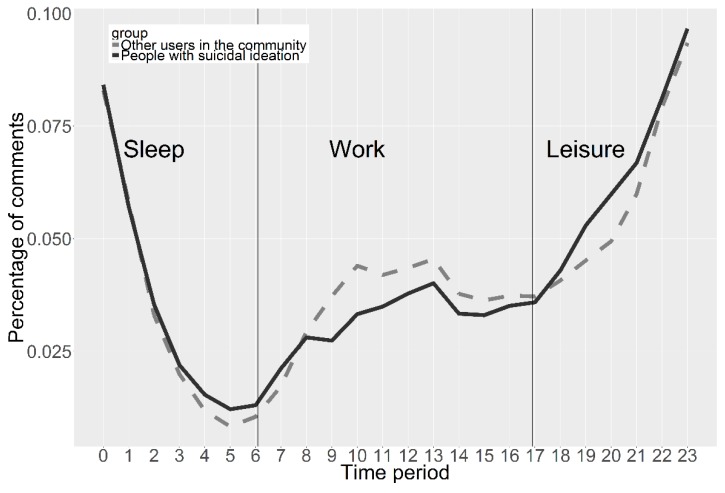
Time distribution of comments.

In addition, the diurnal pattern of PSI’s postings was analyzed in comparison with other users in the community (Figure 4). The results suggest that users of the community may be more active at night.

## 4. Discussion

In this study, we identified 3039 PSI in a Chinese suicidal community on Weibo and explored their behavior in the community. The finding that the agglomeration behavior of these PSI compared with the findings from previous study (114 PSI detected from 1 million Weibo users [37]), highlights the importance of suicide prevention at community level. To the knowledge of the researchers, this is the first study to report the behavior of PSI at community level.

Through content analysis, it was found that past suicide attempters in the community, who reported little fear of death [57], may still want to commit suicide and propagate their suicide methods in a painless way. Their postings may affect vulnerable people who are willing to search for painless suicide methods to overcome their inner fear [58,59]. That is, PSI in the community (e.g., users who are afraid of suicide and those who are looking for suicide methods) may be at risk of suicide due to the copycat effect [60,61,62]. Our findings suggest that additional support should be provided for users with previous suicide attempts, and it is necessary for social web sites to curb the spread of suicide methods in the community, which may reduce the risk of copycat suicides and the possible contagion effect of suicides.

By learning the interactional patterns of PSI, it was found that the more times users communicate with PSI, the more likely that users become suicidal. This phenomenon is in line with previous studies that interpersonal communication may lead to suicide contagion [31,56]. We also found that PSI who are involved in the contagion process are more likely to find someone to commit suicide with them than other PSI. The underlying mechanism has not been examined. One possibility is that these PSI want to get acquainted with someone (who are similar to them) to supervise each other in the process of committing suicide [45]. More research is required to investigate the reason behind this finding. Future work will focus on the details of communication (e.g., what do they talk about, how their inner feelings change, why do these users like to communicate in the online community, and what kind of communication can alleviate their suicidal ideation) among these users to promote the effectiveness of suicide prevention.

From the active patterns of PSI, we found that PSI are significantly more active in the online community. One possible explanation of this phenomenon may be that they hope to be accepted by other PSI in the community [45]. However, more research is required to determine why they are more active (especially at night) in the community. This finding may improve our understanding of PSI’s behavior in suicidal communities. Considering the fact that the online behavior of PSI may influence the effectiveness of online suicide prevention [63,64], targeted suicide prevention programs should be implemented to support individuals who show a different attitude towards death in the community. For instance, delivering feelings of fear and pain to vulnerable people in the community (e.g., individuals who are biologically frightened of death) could possibly be the most effective way to intervene and prevent suicides [65,66].

Similar to findings of previous studies that Chinese women have a higher suicide rate than men [6,67], we also found that more than 75% of PSI were female. This gender difference may be related to the specific characteristics of Chinese culture and social environment (e.g., self-esteem, family values and social status [67,68]). For example, Chinese women may be typically blamed for different types of family disputes, and which will make them feel that they are not supported by others in their family network and in society [67,69]. Considering the higher prevalence of suicide ideation and suicide attempts of females [70,71], our findings suggest that gender differences should be considered in implementing effective prevention strategies.

Nevertheless, it is important to notice the limitations of the current study. Firstly, it should be noted that the method used in this study is not a substitute for traditional clinical diagnosis. It is, however, an effective complement to the traditional methods because of its low cost in identifying and assisting PSI. This sample is limited to those individuals who self-reported their suicidal ideation. There are certain biases inherent in self-reporting methods, and future research should determine whether results could be replicated when interview and other clinical approaches are employed. Secondly, our findings may have limited generalizability since the sample may be different to samples from other countries due to cultural differences. Though the findings contribute to an under-researched area, further research should be conducted among different populations. Thirdly, our study considered communicating with PSI as contributing to the spread of suicidal ideation. Although our work proved a positive correlation between the times users communicate with PSI and the probability that users will become PSI, further work is needed to study how suicidal ideation spreads during communication among users. Fourthly, as our data is obtained from the public community, it is important to note that the behavior of PSI in a private community may be different from our results.

## 5. Conclusions

Despite these limitations, this study represents the first social media study on the behavior of PSI in Chinese suicidal communities. By investigating their behavior revealed in the community, our study stresses the importance of suicide prevention in suicidal communities on social media, especially for those individuals who might not seek mental health services. In addition, our findings highlight the necessity of curbing the spread of suicidal ideation, methods and behaviors, which have been observed in diverse cultures [72,73,74].

## Figures and Tables

**Table 1:** Inter-coder reliability for the five themes.

Themes	Kappa
Expression of suicidal ideation	0.96
Inquiry of suicide methods	0.91
Expression of thanatophobia	0.93
Description of suicide attempts	0.86
Seeking suicide partners	0.99

**Table 2:** Themes of PSI’s suicidal comments.

Themes	Suicidal Comments (*n* = 4489)	Example Postings
Expression of suicidal ideation	3300 (73.5%) ^1^	I also want to commit suicide!
Inquiry of suicide methods	263 (5.9%)	How can I commit suicide without pain?
Expression of thanatophobia	513 (11.4%)	I want to commit suicide, but I’m afraid of pain.
Description of previous suicide attempts	117 (2.6%) ^1^	I tried to kill myself, but did not succeed. The pills I eaten were not sufficient.
Seeking suicide partners	304 (6.8%)	@somebody: There is no meaning to be alive. I will commit suicide next Saturday, can you come with me?

^1^ There were 8 comments simultaneously recorded in these 2 themes, in which PSI not only described their previous suicide attempts, but also desired to suicide again.

**Table 3:** The suicidal themes mentioned by the two groups.

Themes	Suicidal Comments by PSI Involved in Spreading Suicidal Ideation (*n* = 499)	Suicidal Comments by other PSI (*n* = 3990)	χ^2^	*p*
Expression of suicidal ideation	307 (61.5%)	2993 (75.0%)	40.8	<0.001
Inquiry of suicide methods	45 (9.0%)	218 (5.5%)	9.5	0.002
Expression of thanatophobia	51 (10.2%)	462 (11.6%)	0.68	0.4
Description of previous suicide attempts	15 (3.0%)	102 (2.6%)	0.19	0.65
Seeking suicide partners	81 (16.2%)	223 (5.6%)	77.9	<0.001

**Table 4:** Demographic characteristics of PSI.

Variable	PSI ^1^ in the Community	Other Users in the Community	Cohen’s d	*p*
	(*n* = 3039)	(*n* = 164,466)		
**Gender, %**				<0.001
Male	25	35		
Female	75	65		
**Followers, *n***			0.0002	<0.001
Quartile 1	73	124		
Median	239	322		
Quartile 3	656	747		
**Following, *n***			0.0001	<0.001
Quartile 1	74	125		
Median	189	261		
Quartile 3	388	464		
**Postings, *n***			0.0003	<0.001
Quartile 1	32	97		
Median	199	537		
Quartile 3	930	1877		

^1^ people with suicidal ideation.

**Table 5:** Active patterns of users in the community.

Deadline	PSI in the Community		Other Users in the Community	
	Total Number	Active Number	Total Number	Active Number
	*n*	*n* (%)	*n*	*n* (%)
September 2012	453	58 (12.8)	79,488	1300 (1.6)
March 2013	649	91 (14.0)	95,449	1606 (1.7)
September 2013	859	110 (12.8)	107,456	1887 (1.8)
March 2014	989	133 (13.4)	112,677	2013 (1.8)
September 2014	1191	173 (14.5)	118,999	2216 (1.9)
March 2015	1360	201 (14.8)	127,651	2516 (2.0)
September 2015	1715	255 (14.9)	133,723	2689 (2.0)
March 2016	2045	300 (14.7)	139,837	2898 (2.1)
September 2016	2534	366 (14.4)	155,224	3306 (2.1)
February 2017	3039	477 (15.7)	164,466	3743 (2.3)

There was a statistically significant difference (*p* < 0.001) between the two groups in each set of the data.
